# Results from “Live Well”, a randomized controlled community-based participatory intervention to prevent obesity in new immigrant mother–child dyads

**DOI:** 10.1186/s12889-023-16727-z

**Published:** 2023-10-02

**Authors:** Christina D. Economos, Alison Tovar, Silvina Choumenkovitch, Rebecca Boulos, Kenneth Chui, David M. Gute, Raymond R. Hyatt, Nesly Metayer, Alex Pirie, Aviva Must

**Affiliations:** 1 Gerald J. and Dorothy R. Friedman School of Nutrition Science and Policy, Tufts University, Boston, MA USA; 2 Department of Behavioral and Social Sciences, Center for Health Promotion and Health Equity, Brown University School of Public Health, Providence, RI USA; 3 Maine Public Health Association, Augusta, ME USA; 4 Department of Public Health and Community Medicine, School of Medicine, Tufts University, Boston, MA USA; 5 Department of Civil and Environmental Engineering, School of Engineering, Tufts University, Medford, MA USA; 6 Freeport Development Strategies, Burlington, MA USA; 7 Center for Public Management, Suffolk University, Boston, MA USA; 8 Immigrant Service Providers Group/Health, Somerville, MA USA

**Keywords:** Immigrants, Obesity, Randomized controlled trial, Community-based participatory research

## Abstract

**Background:**

Upon arrival, the prevalence of overweight and obesity is lower in new immigrants than their native counterparts in the U.S. With longer residency in the U.S., these differences converge over time, followed by higher prevalence among immigrants than native U.S. residents. Results from the *Live Well* project in the Greater Boston area demonstrate the viability of utilizing a culturally adapted, community-based participatory research (CBPR) approach to reduce weight gain among newly immigrated mother–child dyads.

**Methods:**

Haitian, Latina, and Brazilian mother–child dyads (*n* = 390), new to the U.S. (fewer than 10 years) were enrolled in a one- to two-year long CBPR lifestyle intervention that targeted dietary and physical activity behaviors. Attendance was recorded to establish dose. Demographics, anthropometrics, and relevant covariates were collected from participants at baseline, 6, 12, 18, and 24 months. Body Mass Index (BMI) was calculated using objectively measured height and weight. Linear mixed regression models were used to assess change in BMI and BMI z-score of mothers and children respectively.

**Results:**

At baseline, nearly 75% of mothers and 50% of children were either overweight or obese (BMI ≥ 25.0 and BMI z-score ≥ 85^th^ percentile, respectively). Only 20% of mothers attended all 12 intervention sessions in year 1. Using intent-to-treat analyses, no significant time, intervention, or time × intervention effects were observed for weight change of mothers or children at follow-up. Mothers in the highest quantile (those who attended all 12 intervention sessions) had significant reductions in BMI at 18 months (1.76 units lower, 95%CI: -3.14, -0.37) and 24 months (2.61 units, 95%CI -3.92, -1.29) compared to mothers in the lower quantiles, including those with no exposure. Such dose effects on BMI z-scores were not noted for children.

**Conclusions:**

Findings from *Live Well* demonstrate the viability of utilizing a CBPR approach to address overweight and obesity among immigrant mothers. Given the higher-than-expected prevalence of overweight and obesity among mother–child dyads by ~ 6 years of U.S. residency, and lower maternal participation rates in the intervention, additional research is necessary to identify the optimal intervention length, retention strategies, and approach to jointly support healthy maternal and child weight.

## What is already known about this subject?

- The United States has a larger foreign-born population than any country and nearly one in five (19%) Americans will be foreign born by 2050. Yet, they are underrepresented in the current health literature and are disproportionately at risk for overweight and obesity.
- Interventions to prevent obesity among new immigrants must be delivered early in the acculturation process, given the known associations of length of U.S. residency and weight gain.
- Studies should employ community-based participatory approaches to successfully recruit and retain participants and engage community organizations that serve new immigrants.

## What does our study add?

- Few community-based, randomized controlled trials have been developed for new immigrant mothers and children. *Live Well* is among the largest and longest trials to reach this under-studied and under-resourced population using participatory methods to develop and implement a culturally appropriate intervention.
- This study demonstrates that a culturally adapted, community-based intervention can successfully reduce Body Mass Index (BMI) in new immigrant mothers in a dose–response relationship over 2 years.

## Introduction

The United States (U.S.) has more immigrants than any other country in the world; more than 1 million immigrants arrive in the U.S. each year and their descendants are projected to account for 88 percent of the population growth in the U.S. [1]. Consistent with the immigrant epidemiological paradox, new immigrants have lower average body mass indices (BMIs) and longer life expectancies than U.S.-born counterparts upon arrival [2–4]. However, their risk of diet-related chronic diseases increases with longer U.S. residency time [4–13].

Rapid weight gain and resulting disproportionate rates of overweight and obesity have been documented among immigrants who arrive to the U.S. from various countries [14]. Findings from the New Immigrant Survey [14] indicate that immigrants with longer U.S. residency (10 years or more) have higher levels of overweight and obesity than the population average and these trends persist across various ethnic groups. These patterns were also noted for children; Haitian-immigrant children have a lower initial prevalence of overweight and obesity compared to U.S.-born peers (30% and 51%, respectively) and their BMI percentile increases by 3.7% per year of U.S. residency [15]. Similarly, compared to adults residing in Mexico, Mexican immigrants residing in the U.S have elevated rates of overweight and obesity [16–18].

Immigrants often experience a combination of economic, educational, linguistic, and social stressors, including a lack of access to essential health and social services, after they arrive in the U.S. To cope with these stressors, along with limited access to affordable healthy foods, insufficient time for meal preparation and physical activity, and exposure to “obesogenic” food environment [5, 6, 19, 20], new immigrants modify their diet and physical activity behaviors significantly during the first 10 years of U.S. residency [21, 22]. The lifestyle modifications contributing to the shift from initial immigrant health advantage to subsequent health disadvantage [6, 11, 19, 23–25] highlight the need for appropriately-timed prevention efforts within the multidimensional process of acculturation [26].

Despite the well-documented risk of diet-related chronic diseases, few weight-related interventions have successfully focused on new immigrants [27] or engaged with an immigrant community early in the immigration process to identify how to best intervene [27]. Given current projections for new immigrants to account for 19% of the U.S. population growth by 2050 [1], reaching this population with culturally-adapted obesity prevention interventions is a public health priority.

Evidence-based, community-based, culturally adapted approaches present a promising opportunity to halt the rising prevalence of overweight and obesity within the increasing U.S. immigrant population. *Live Well* was created by a diverse group of researchers and community partners, to assess whether an appropriately timed intervention developed with active input from community partners and the immigrant community could be successful at preventing unhealthy weight gain among new immigrant (≤ 10 years in the U.S.) mothers and children in the Greater Boston area. This paper examines the efficacy of the *Live Well* intervention on the weight trajectory of new immigrant mother–child dyads residing in the Greater Boston area.

## Methods

### Study design and setting

*Live Well* was a randomized controlled intervention (2008–2012) intended for the largest new immigrant groups in the Greater Boston, MA area from Brazil, Latin America, and Haiti. The intervention was developed using a community-based participatory research (CBPR) approach and was designed and implemented by a culturally and professionally diverse steering committee formed at the onset of the project [28]. Throughout recruitment, process data were collected to help inform and adjust ongoing recruitment efforts. The one-year *Live Well* curriculum incorporated information about key nutrition and physical activity topics and was delivered by project coordinators in a structure that facilitated the sharing of participant experiences and group discussions around behavior change. In addition to the group sessions, participants also received five individual phone calls from each of the project coordinators, where motivational interviewing techniques were used to discuss priorities and goals set during the group sessions. Based on input from community partners and lessons learned from the first year of curriculum implementation, the community partners developed and implemented a second-year civic engagement curriculum which included six group sessions on community engagement to address social, environmental, and policy issues that impact obesity. Eligible participants were recruited into two separate cohorts over two years of recruitment (cohort 1 Spring 2010; cohort 2 Spring 2011) and randomized to an intervention or control group [29]. The civic engagement extension was offered to intervention women in cohort 1 only. A delayed intervention was offered to participants in the control group at the end of the study. Descriptions of the curriculum development for the intervention, participant recruitment, informed consent, baseline characteristics; feeding style and child weight; feeding style and evening meals; as well as occupational physical activity and weight-related outcomes are published elsewhere [28–36]. All outcome measures were collected at neighborhood community organizations. All aspects of the *Live Well* study were approved by the Tufts University Institutional Review Board.

### Study participants

Mothers immigrating from Brazil, Latin America, and Haiti were eligible for the *Live Well* intervention. The mother–child dyads met inclusion criteria if the mothers lived in the Greater Boston area, were not pregnant (or more than 6 months postpartum), between the ages of 20–55 years, had a child between the ages of 3–12 years, and were willing to be randomized. Informed consent was obtained from all participants, assent was obtained for all children over 7 years of age, and written consent for children under 7 years of age was obtained from a caregiver.

## Measurements

### Primary outcome: anthropometric measurements

Height and weight measurements were taken in triplicate (or until three measurements were within ± 0.25 cm and 0.5 pound (lb.), respectively) and averaged, following standardized procedures, and used to calculate BMI (mothers) and BMI z-score (children), the primary outcomes of the study [37, 38]. Height was measured without shoes to the nearest eighth of an inch using a portable Shorr Board vertical stadiometer (Shorr Production, Olney, MD). Weight was measured in light clothing, without shoes, to the nearest 0.5 lb. on a portable digital scale (Befour PS-6600 Portable Scale; Befour Inc., Saukville, WI). Weight status was classified based on BMI (mothers) or BMI z-score (children) as recommended by the U.S. Centers for Disease Control and Prevention [39], as follows – BMI: underweight (< 18.5), normal weight (18.5–24.9), overweight (25.0–29.9) and obese (≥ 30.0); BMI z-score: underweight (≤ 4.5^th^ percentile), normal weight (5^th^-84.5^th^ percentile), overweight (85^th^-94.5^th^ percentile), and obese (≥ 95^th^ percentile).

### Covariate measures

Additional variables used for analyses were collected through self-administered (or interpreter-assisted) surveys completed by the participants (mothers) at baseline and on follow-up measurement days. The survey included questions about socio-demographic characteristics including maternal age, race/ethnicity, marital status, education, and occupation. Mothers’ perceived level of acculturation was assessed through survey questions, including mother’s country of birth, home setting (urban or rural), number of years (or months) living in the U.S., and reasons for moving to the U.S. Perceived level of acculturation was rated using the following statement: “When you think about your daily life now, where would you place yourself?” Participants answered using a 10-point Likert scale (1 being “More American” and 10 being “More Brazilian/Haitian/Latino”). Behavioral measures were also collected and reported elsewhere [28–36]. The survey also asked about the participating child’s race/ethnicity, age, and gender. Project staff recorded attendance at group sessions and individual phone calls to estimate intervention dose.

### Statistical analyses

Descriptive statistics of demographics were derived by program status and then tabulated. Mean and standard deviation (SD) of the main outcomes: maternal BMI and child’s BMI z-score, by program status and time point were also tabulated. Linear mixed regression models were used to isolate the adjusted association between intervention and the outcome of interest. The differences in the trend of the outcomes were assessed along three time points: baseline, 6, and 12 months. To capture the impact of the second-year intervention, trends were also examined over two additional time points: 18 and 24 months.

#### Sensitivity analysis on exposure dosage

There was a great deal of variation in attendance (level of exposure). To address this, we introduced a set of sensitivity analyses to examine possible effects due to different levels of exposure in three forms: i) Binary form—participants assigned to the intervention who never attended any session were coded as non-exposed (0), together with those assigned to the control; participants assigned to the intervention who had attended at least one session were coded as exposed (1); ii) Quantile form— participants assigned to the intervention who never attended any session were coded as non-exposed (0), together with those assigned to the control; participants in the intervention group who attended at least one session were coded as having level 1 exposure: 1–4 sessions; level 2: 5–7 sessions; level 3: 8–11 sessions; and level 4: all 12 sessions; and iii) Continuous form—exposure was represented by the number of sessions attended.

The overall program effects were examined by jointly testing the interaction between time point and exposure. The same protocol was implemented to assess the primary outcomes of maternal BMI and child BMI z-score. Given that three different ways were used to assess exposure, a Bonferroni adjustment was applied and a threshold of *p* < 0.017 (0.05 divided by 3) was used to determine statistical significance. The statistical test results for the program effects were tabulated. Regression results were visualized by plotting the predicted margins at each time point for each exposure category.

#### Equity of program effect

Two exploratory models were developed to examine if the intervention effects were equitable across immigrant group and BMI categories (underweight, normal weight, overweight, and obese.) We included a set of 3-way interactions between i) intervention assignment × time × immigrant group; and ii) intervention assignment × time × BMI category and tested their joint significance. For all equity investigations, exposure was modeled as intent-to-treat.

#### Covariates

For the maternal models, covariates included mother’s age and BMI at baseline; for the child models, covariates included child’s age and BMI z-score at baseline. Both models adjusted for length of stay (months) in the U.S., maternal education (high school or above), and immigrant group (Brazilian, Latina, or Haitian).

All analyses were performed using Stata 14 (StataCorp, College Station, TX). A priori type I error rate was set at 5.0% for the planned analyses, and 1.7% (5.0% divided by 3) for the sensitivity analysis to adjust for the three different exposure forms that were tested. Participants’ unique identification number was included as a random intercept to account for individual-level clustering; exchangeable covariance structure was used for the mixed-effects model.

##### Sample size

To obtain the largest sample based on the literature, we used conservative estimate of 1.0 kg weight gain in the control group and weight loss/no weight gain in the intervention group, with a standard deviation of 3.0 kg. The test of equality of means was carried out at the 0.050 level of significance. A sample size of 145 women per group (intervention and control) gave a 0.80 probability of rejecting the null hypothesis of equal means. To account for drop out and lost to follow up, the sample was increased by 35%.

## Results

### Participant characteristics

**Fig. 1:**
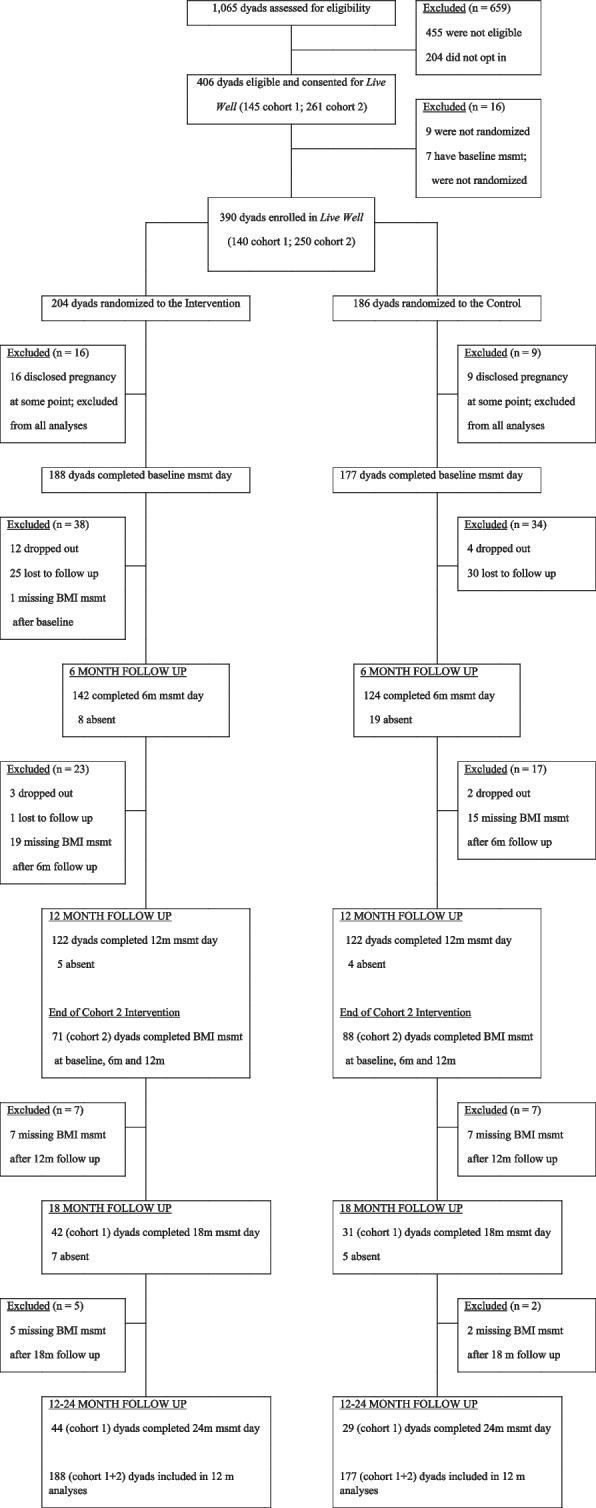
The participant flow diagram, stratified by cohort and intervention assignment

Participant recruitment spanned from 2009–2011 and the intervention spanned from 2010–2012. As shown in Fig. 1, of the 390 dyads that were randomized into treatment and control groups, 25 were excluded from analyses due to a disclosed pregnancy during the intervention; 365 were included in the final analyses (188 intervention; 177 control). Attendance at follow-up measurement varied by time-point and intervention status, with better overall attendance in the intervention compared to the control.

Overall, no significant differences were observed between the intervention and control group at baseline on any maternal or child characteristics (Table 1). On average, the mothers were 35.9 (intervention) and 36.6 (control) years of age and children were 6.2 (intervention) and 6.4 (control) years of age. Mothers had resided in the U.S. for about 6 years for both intervention and control groups, and about two-thirds (75 percent and 65.7 percent in the intervention and control group, respectively) had completed high school. At baseline, about three-fourths of the mothers (75 percent and 72.1 percent in the intervention and control group, respectively) and slightly less than half (45.7 percent in the intervention and 44.5 percent in the control group, respectively) of the children were classified as overweight or obese.

**Table 1:** Baseline demographic characteristics of mothers and children participants in *Live Well* according to intervention assignment (*n* = 364 mother–child dyads)^a^

	Intervention		Control	
	N		N	
***Mother***				
N	187	-	177	-
Age, year (SD)	35.9	(6.5)	36.6	(6.4)
Race/ethnicity (N, %)				
Brazilian	69	36.9%	55	31.1%
Haitian	63	33.7%	66	37.3%
Latino	55	29.4%	56	31.6%
Time (years) residing in US (SD)	6.3	(3.3)	6.1	(3.2)
Education ≥ High School (N, %)	128	70.0%	115	65.7%
Baseline BMI (SD)	30.0	(6.0)	29.0	(5.6)
Overweight/Obese (N, %)	138	75.0%	127	72.1%
***Child***				
Age, year (SD)	6.2	(2.7)	6.4	(2.8)
Baseline BMI-Z (SD)	0.94	(1.2)	0.87	(1.1)
Overweight/Obese (N, %)	84	45.9%	77	44.5%

^a^Due to missing data, sums of percentages may not add up to 100%

**Fig. 2:**
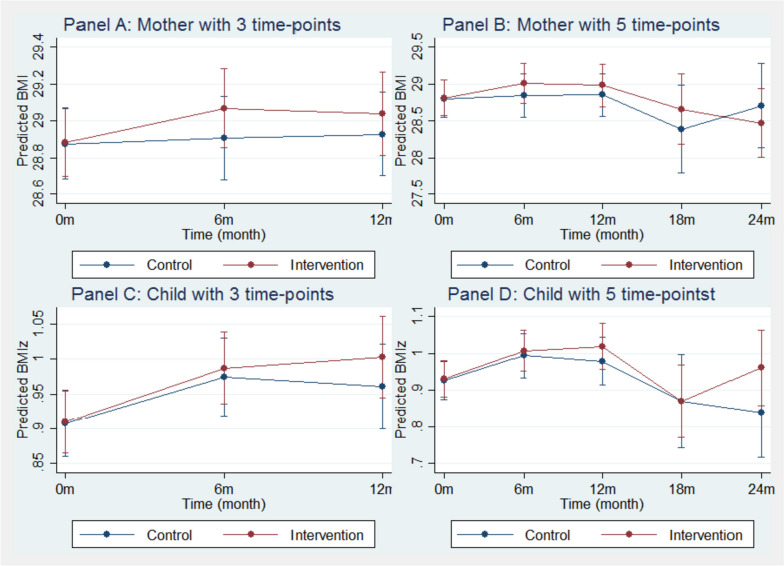
Predicted margins of the crude and adjusted regression analyses using randomization results as the exposure indicator

There was no significant effect of the intervention on maternal BMI or children’s BMI z-score. (Table 2 shows coefficients and Table 3 shows the joint significance test of the program effects). In the maternal models, baseline BMI was the only significant predictor of higher BMI at follow-up (12 months or three time points and 24 months or five time points). In the child models, baseline BMI z-score and age were significant predictors of higher BMI z-score at follow-up (12 months and 24 months). The predicted margins for these four models are plotted in Fig. 2; as seen, no-significant differences were observed for either mothers or children across the 5 time-points (Panels A, B, C, D – Fig. 2).

**Table 2:** Regression results of the crude and adjusted analyses using randomization results as the exposure indicator

	**Outcome: Mother’s BMI**						**Outcome: Child’s BMI z-score**					
	**3 time-points**			**5 time-points**			**3 time-points**			**5 time-points**		
	**β**	**95%CI**	***p***	**β**	**95%CI**	***p***	**β**	**95%CI**	***p***	**β**	**95%CI**	***p***
**Intervention**	.008	-.262, .278	.954	.012	-.336, .359	.948	.003	-.062, .068	.931	.005	-.066, .076	.886
**Time**												
**Baseline**	Ref	-	-	Ref	-	-	Ref	-	-	Ref	-	-
**6**^**th**^** month**	.032	-.237, .301	.816	.045	-.287, .376	.792	.067	**.001, .134**	**.048**	.067	-.001, .136	.054
**12**^**th**^** month**	.053	-.215, .322	.696	.051	-.280, .382	.761	.053	-.017, .124	.138	.053	-.020, .125	.156
**18**^**th**^** month**	-	-	-	-.419	-1.04, .202	.186	-	-	-	-.057	-.188, .075	.399
**24**^**th**^** month**	-	-	-	-.096	-.687, .495	.750	-	-	-	-.086	-.213, .041	.184
**Intervention × time**												
**Baseline**	Ref	-	-	Ref	-	-	Ref	-	-	Ref	-	-
**6**^**th**^** month**	.153	-.217, .524	.418	.150	-.306, .607	.518	.010	-.082, .101	.837	.008	-.086, .102	.865
**12**^**th**^** month**	.103	-.275, .481	.593	.119	-.346, .585	.615	.039	-.058, .137	.431	.034	-.066, .135	.502
**18**^**th**^** month**	-	-	-	.266	-.528, 1.06	.511	-	-	-	-.004	-.182, .163	.960
**24**^**th**^** month**	-	-	-	-.246	-1.01, .519	.529	-	-	-	.115	-.051, .281	.174
**Age at baseline**^**a**^	-.000	-.017, .017	.996	-.009	-.030, .013	.438	**-.011**	**-.020, -.002**	**.019**	**-.012**	**-.022, -.002**	**.025**
**Outcome at baseline**	**.960**	**.942, .978**	**< .001**	**.938**	**.915, .961**	**< .001**	**.931**	**.909, .952**	**< .001**	**.922**	**.897, .946**	**< .001**
**Months in the US**	-.001	-.005, .002	.415	-.003	-.007, .002	.224	-.001	-.001, .000	.149	-.001	-.001, .000	.212
**High school (Mother)**	-.134	-.353, .085	.229	-.213	-.500, .073	.145	-.020	-.073, .033	.453	-.029	-.088, .031	.348
**Race/ethnicity**												
**Brazilian**	Ref	-	-	Ref	-	-	Ref	-	-	Ref	-	-
**Haitian**	-.084	-.390, .222	.590	-.019	-.416, .378	.927	-.037	-.107, .033	.302	-.010	-.089, .068	.795
**Latino**	.114	-.139, .368	.378	.106	-.223, .435	.529	.016	-.044, .077	.599	.036	-.031, .104	.295
**Intercept**	1.340	0.530, 2.150	< .001	2.407	1.35, 3.46	< .001	.195	.086, .303	< .001	.197	.075, .318	.001

^a^Mothers’ and child’s ages (in years) were used in their corresponding models

**Table 3:** Summary of interaction tests examining program effects with three exposure measures and equity of program effect across race/ethnicity as well as weight categories

		**Mother**		**Child**	
		**3 time-point**	**5 time-point**	**3 time-point**	**5 time-point**
**Program effect**^a^	**Unadjusted**^d,h^	0.732	0.777	0.885	0.849
	**Adjusted**^c,d,h^	0.704	0.799	0.728	0.689
	Binary exposure^c,e,i^	0.210	0.108	0.198	0.279
	Quantile exposure^c,f,i^	0.441	**0.010**	0.465	0.059
	Continuous exposure^c,g,i^	0.138	0.018	0.118	0.037
**Equitable effect**^b^	**Race/ethnicity**^c,d,h^	0.788	0.631	0.590	0.421
	**BMI category**^c,d,h^	0.251	0.846	0.513	0.344

Bolded *p*-value indicates statistical significance

^a^Joint *p*-values for the interaction terms (program × exposure)

^b^Joint *p*-values for the interaction terms (program × exposure × race/ethnicity) & (program × exposure × BMI category)

^c^Adjusted for mother’s age and BMI at baseline, number of months residing in the US, maternal education reached high school or above, and race/ethnicity (Brazilian, Haitian, and Latino) in Mother regression models; adjusted for child’s age and BMIz at baseline, number of months residing in the US, maternal education reached high school or above, and race/ethnicity in Child regression models

^d^Exposure modeled as the results of randomization

^e^Exposure modeled as 0 if in control group or never attended any session; 1 if ever attended any session

^f^ Exposure modeled as 0 if in control group or never attended any session; 1–4 according to the level of exposure split by g quartile if every attended any session

^g^Exposure modeled as number of sessions attended

^h^Significant if *p* < 0.05

^i^Significant if *p* < 0.017, Bonferroni adjustment based on three alternate exposure definitions

**Fig. 3:**
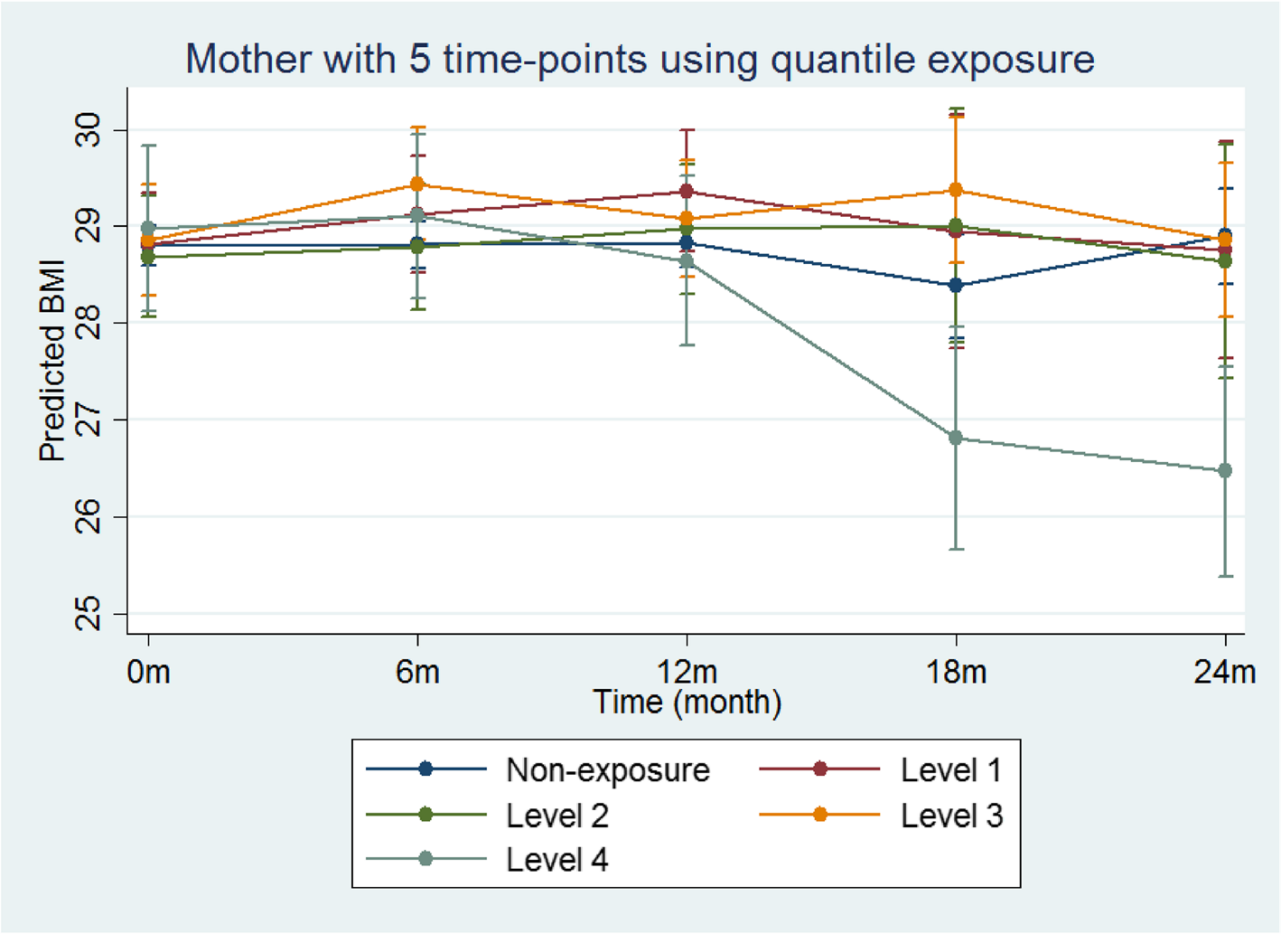
Predicted margins of the regression model predicting mother’s BMI across five time-points with quartiles as the indicator of exposure

When program effects were tested using binary, quantile, or continuous exposure, significant association was noted only for mothers using the quantile exposure measure and five time-point assessment period (Table 3, *p* = 0.010 with threshold of 0.017 after Bonferroni’s adjustment). Mothers who participated in all 12 sessions (~ 20%) had a significantly larger reduction in BMI at 18 months and 24 months compared to mothers in the other exposure groups, including those who were in the weight-listed control group (Fig. 3). While a similar trend was seen when using the continuous exposure measure, the difference did not reach statistical significance (Table 3, *p* = 0.018 with threshold of 0.017 after Bonferroni’s adjustment). We did not observe any differentiation in program effect across immigrant group or by BMI (Table 3, p ranges from 0.42–0.79) and BMI category (p ranges from 0.25–0.51).

## Discussion

The *Live Well* study was developed to promote healthy weights among newly immigrated mothers and their children by offering a culturally relevant and participatory lifestyle intervention. Using intent-to-treat analyses, program participation was not significantly associated with a significant reduction in BMI or BMI-z in mothers or children. However, significant reductions in BMI were noted among mothers who received the full intervention dose at 18- and 24-months versus those with lesser or no attendance (controls).

Our findings are consistent with those noted in other randomized controlled obesity prevention trials among new immigrant populations; those trials also reported that the intervention overall did not achieve significant long-term reductions in BMI (mothers or children) and BMI z-score (children) [40–46]. For example, findings from implementing a culturally-tailored, group-based, lifestyle intervention for 223 Latina immigrants led to improvements in dietary habits and physical activity levels, however, this did not translate to significant reductions in BMI after the intervention or 3-month follow-up compared to controls [43]. Similarly, while a modified curriculum comprised of 6-sessions for 1,104 Hispanic women in the Expanded Food and Nutrition Education program (EFNEP) showed significant BMI reductions at the first post measurement (2 months), this trend did not hold after 4-months [47].

Other trials focused on obesity prevention among children of immigrants have also not been successful in impacting children’s BMI z-score. For example, Hull et al.’s culturally-tailored obesity prevention intervention, delivered to Hispanic families via group sessions at a community center, saw no changes in weight status for Hispanic children [42]. While some studies reaching analogous populations were successful, these studies often focused on child BMI through the delivery of parent health-behavior interventions and did not include parent BMI as an outcome of interest [48–50].

Our finding that only adult participants who received the highest dose were the ones to have a significant impact on BMI, is consistent with the literature. In one 6-month community-based trial, Latina women who attended 7-or-more of 8 nutrition education classes and coaching sessions experienced significantly greater BMI reductions compared to women who attended less frequently [43]. While higher attendance in community-based obesity prevention interventions has been associated with health-promoting behaviors, including higher fruit and vegetable consumption in adults [45] and children [51] and increases in daily physical activity for adults [46], these studies did not observe statistically significant changes in body weight according to intervention assignment, and cited attendance as a key barrier to implementation.

*Live Well* builds upon the existing literature in outreach and intervention duration. *Live Well* is one of the largest and longest duration randomized controlled trials focused on new immigrant communities. It also used an authentic participatory research approach, with community and academic partners sharing responsibility and decision-making about intervention conceptualization, recruitment, implementation, analysis, and interpretation, and including incorporating a process evaluation throughout the program. Compared to existing studies, we recruited a relatively new and diverse population of immigrants from Brazil, Latin America, and Haiti, while previous studies have predominantly enrolled Hispanic or Latino communities with longer residency in the U.S. and held shorter intervention periods [27]. Additionally, the *Live Well* curriculum engaged mothers and by design, their children, in a one or two-year randomized controlled trial, while other studies have primarily focused on either adults or children [27]. Other interventions engaging parent–child dyads have had shorter curriculum durations (3–6 months) as well as smaller sample sizes (160 dyads, or fewer). Weight loss prevention trials prior to *Live Well* have focused on communities with 10–20 years of U.S. residency [43, 52, 53]; *Live Well* successfully recruited relatively new immigrants – with an average residency in the U.S. of 6.3 years.

Although the first 10 years of U.S. residency are often considered a critical period for change, findings from *Live Well* highlight the importance of early prevention intervention efforts, given higher than expected prevalence of overweight and obesity among new immigrant mothers at baseline (71.5%) with only ~ 6 years of U.S. residency and the well-documented relationship between U.S. residency and dietary changes, lower physical activity levels, as well as higher average BMI [7]. Reversing these unhealthy lifestyle behaviors and the incumbent weight gain is particularly difficult [8, 9], as evidenced by our observations that higher baseline BMI and BMI z-score were significantly predictive of elevated weight status at follow-up.

Lessons learned from *Live Well* provide evidence that higher dose intervention participation is associated with reduction in maternal BMI over time and additional strategies to successfully retain a high-risk population of mothers and children are required. Although we recruited a diverse group of new immigrants and implemented a successful two-year intervention, our efforts were not without barriers and limitations. First, new immigrants are a hard-to-reach community who are often hesitant to trust unfamiliar research institutions. As previously described, *Live Well* did benefit from previous collaboration between the community-based organizations and the research team [28]. Additional recruitment barriers include linguistic barriers, time constraints, misunderstanding of study protocol, and concern with sharing legal documentation status. To overcome these challenges, we adjusted our initial eligibility criteria – expanding the “new immigrant” definition from ≤ 5 to ≤ 10 years U.S. residency and revised the study design – shortening the *Live Well* curriculum from two years to one for cohort 2 and introducing a one-year civic engagement extension for cohort 1. It is likely these adjustments limited our ability to achieve significant impact, overall. The expanded definition allowed immigrants with longer U.S. residency and higher degrees of acculturation to participate, which may explain the higher-than-expected prevalence of obesity at baseline. Low and varied attendance may have further limited the dose and quality of the intervention: only 20 percent of mothers attended all sessions and completed all measurement days. Our finding that mothers who participated fully in the intervention had significant improvement in BMI is encouraging and suggests the potential for future intervention impact.

Barriers to participation included working multiple jobs [36], time stress [31, 36], and acculturation stress [36]. Furthermore, participant compensation was limited by grant funding. To improve participant engagement and retention, future studies should consider using a range of communication channels such as text messages, social media, virtual meetings, and organizing social events with a health focus. Additional research is needed to expand ways to fully engage and retain new immigrants in their early years into the U.S., including ways to engage them in steering committees, testing various modes of delivery, duration of program, and integration as well as coverage of topics that are of higher priority to the population. Furthermore, interventions should address and reduce barriers faced by new immigrants, including access to health care and other social safety net programs. To do this, continued investments and cooperation from both the research and intervention communities is needed.

## Conclusion

*Live Well*, a culturally adapted, community-based intervention, is a promising approach for reducing BMI in new immigrant mothers. In consideration of the multiple and complex time, stress, language, isolation, and economic demands experienced by newly arrived immigrants to the U.S., they are considered a “hard-to-reach” study population. The use of modern communication and engagement tools (e.g., social media, texting) and efforts to promote social cohesion and connection may be effective strategies for increasing. Additional research is needed to understand the optimal period to intervene, necessary dose, as well as duration and intensity of the intervention. Future research should include adequate budget and planning time for intended recruitment, sample stratification to capture the diversity of immigrant populations, social support within the program curriculum, and incentives to minimize study attrition.

## Data Availability

The datasets used and/or analyzed during the current study are available from the corresponding author on reasonable request.
